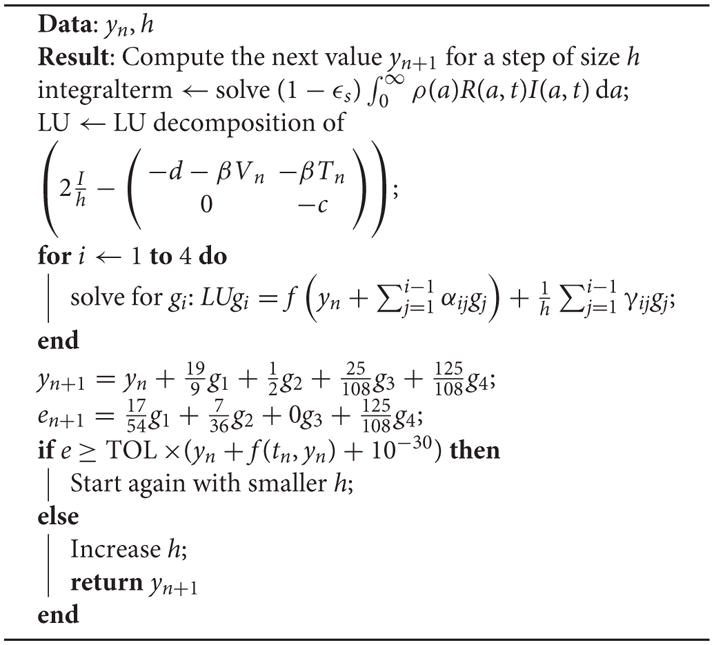# A Robust and Efficient Numerical Method for RNA-Mediated Viral Dynamics

**DOI:** 10.3389/fams.2017.00020

**Published:** 2017-10-31

**Authors:** Vladimir Reinharz, Alexander Churkin, Harel Dahari, Danny Barash

**Affiliations:** 1 Department of Computer Science, Ben-Gurion University of the Negev, Beer-Sheva, Israel; 2 Department of Software Engineering, Sami Shamoon College of Engineering, Beer-Sheva, Israel; 3 Program for Experimental and Theoretical Modeling, Division of Hepatology, Department of Medicine, Loyola University Medical Center, Maywood, IL, United States

**Keywords:** hepatitis C virus, multiscale model, age-structured model, RNA-mediated viral dynamics, partial differential equations, numerical solution, Rosenbrock method

## Abstract

The multiscale model of hepatitis C virus (HCV) dynamics, which includes intracellular viral RNA (vRNA) replication, has been formulated in recent years in order to provide a new conceptual framework for understanding the mechanism of action of a variety of agents for the treatment of HCV. We present a robust and efficient numerical method that belongs to the family of adaptive stepsize methods and is implicit, a Rosenbrock type method that is highly suited to solve this problem. We provide a Graphical User Interface that applies this method and is useful for simulating viral dynamics during treatment with anti-HCV agents that act against HCV on the molecular level.

## 1. INTRODUCTION

Approximately 71 million people worldwide are affected by chronic hepatitis C viral (HCV) infection, which is the primary cause of liver cirrhosis, liver cancer and liver transplant [1]. Approximately 400,000 people die each year from HCV, mostly from cirrhosis and hepatocellular carcinoma [2]. There is no vaccine for HCV and for more than a decade the standard-of-care of pegylated interferon-alpha (IFN) and ribavirin was suboptima [3]. However the recent advent of direct-acting antivirals (DAAs) allows for interferon-free, all-oral treatment yielding cure rates exceeding 90% with pangenotypic activity and shorter durations of therapy (8–24 weeks) compared to IFN-based therapy (24–48 weeks [4]). While these highly effective DAAs are considered one of the greatest achievements in medicine, significant challenges remain for eliminating HCV infection such as finding an optimal approach to current DAA failures, preventing re-infection, identifying all those infected and the high cost of the new DAAs which represents a major barrier to treating the populations that are most affected by HCV [3]. Thus, there exists a continuing need for more affordable therapies, as well as an effective vaccine [5].

Mathematical models are valuable tools for understanding the *in vivo* serum dynamics of viruses that trigger both persistent infection (e.g., HIV-1 [6–9], hepatitis B virus [10–12], hepatitis D virus [13–15], Theiler murine encephalomyelitis virus [16], herpes simplex virus [17] and HCV [18–20]) and acute infection (e.g., influenza A [21–23] and ebola [24]). Mathematical modeling is also improving our understanding of intracellular viral genome dynamics [25–28] and the quantitative events that underlie the immune response to pathogens [6, 9]. The standard model for HCV kinetics during treatment provided many insights into the effectiveness and mechanism of action of IFN and ribavirin (reviewed in [29, 30]). This model has been able to retrospectively predict the duration of treatment needed for HCV eradication (cure) [31–35] and more recently was used in real-time (on treatment) to predict the duration of therapy needed to achieve cure with an IFN-free regimen of silibinin +ribarivin [36]. In the age of DAAs, new models are being developed to meet the challenge associated with these new agents [37–39]. Notably, the first age-based multiscale mathematical model for HCV kinetics has been developed [25, 40, 41] providing a more comprehensive understanding of viral treatment response kinetics observed in patients treated with IFN, HCV protease inhibitors (telaprevir and danoprevir), or the HCV NS5A inhibitor daclatasvir as well as modes of action of these drugs.

The aforementioned model is an extension to the classical Neumann et al. biphasic model [20] that was introduced in 1998 and treated the infected cell as a “black box,” producing virions but without any consideration of the intracellular viral RNA replication and degradation within the infected cell [26, 27, 42]. The biphasic model is a set of three ordinary differential equations (ODEs) with three variables: uninfected target cells (*T*), productively infected cells (*I*), and extracellular virus in blood (*V*). The multiscale model considers the intracellular viral RNA as an additional equation for the variable (*R*), with the introduction of age-dependency and time-dependency, making it a partial differential equation (PDE)model. When this multi scale model is used to study the dynamics of HCV infection under therapy with DAAs it includes both intracellular viral RNA replication/degradation and extracellular viral RNA (i.e., virus particles) with age-dependency and time-dependency. As such, it is considerably more difficult to solve compared to the standard biphasic model. Previously short-term and long-term analytical approximations were derived [25, 41, 43]. In the short-term approximation, it was assumed that after therapy is initiated the infected cells maintain their steady-state levels of HCV, whereas in the long-term approximation all new infections after the onset of therapy are neglected. While the short-term approximation has been shown to be precise only in the first half-day of treatment, the long-term approximation is in agreement after several days post-treatment initiation with a simple numerical solution that utilized a canned solver (an ODE solver used in higher level languages such as Matlab and Mathematica, or Python) [41].

In this paper, we provide a robust and efficient numerical method for solving the multiscale model. The goal is to considerably improve the numerical solution presented in Rong et al. [41] that used a canned solver (an ODE solver used in higher level languages such as Matlab and Mathematica, or Python), making the numerical solution a flexible and robust entity alongside the analytical approximations. As it turns out, because of the properties of this multiscale model and the fact that the differential equations are stiff, some advanced numerical methods that involve adaptive stepsize are needed. To begin with, the use of a canned solver should be replaced with a full-fledged solver because of the additional integral introduced in the multiscale model for the variable *V* that needs to be computed at each time step. Unlike the construction of numerical schemes in other applications, for example in the non-linear diffusion of digital images [44–46] where accuracy can be limited, herein it is adviseable to construct a stable and efficient scheme that belongs to the Runge-Kutta family with at least a fourth order of accuracy. However, due to the nature of the differential equations that are stiff and the additional integral that needs to be evaluated at each time step, implicit solvers with adaptive stepsize are considerably more stable and efficient than the standard Runge-Kutta fourth order method. We implement implicit schemes with adaptive stepsize [47] that are highly efficient and stable for use in the multiscale model with age of hepatitis C virus dynamics.

The main contribution of this manuscript is in the use of the Rosenbrock method to solve the pre-existing multiscale model of hepatitis C virus (a system of PDEs), and to provide an open-access graphical user interference (GUI) to solve and simulate the model numerically. To the best of our knowledge, this is the first numerical simulator with a graphical user interface developed for the multiscale model. In addition, a detailed presentation of the Rosenbrock method is provided, as well as updated results. The paper is organized as follows. Section 2 provides the mathematical background for the model. In section 3, we provide the details of the Rosenbrock method including a step-by-step derivation starting from the standard Runge-Kutta fourth order, describing how we applied it to solve our problem. Section 4 presents and discusses the results achieved with our implementation of the Rosenbrock method, culminating with a description of the multiscale model simulator that we developed and is provided for general use. We conclude the paper with a summary of our results and future work.

## 2. MATHEMATICAL BACKGROUND

### 2.1. The Standard HCV Model

The standard model that has been used and modified for studying hepatitis C viral dynamics is the Neumann et al. model [20]. The three variables this model keeps track of are the target cells *T*, in Equation (1a), the infected cells *I* in Equation (1b) and the extracellular virus *V* in Equation (1c). The target cells *T* are produced at constant rate *s*, and decreased by the number of cells infected by virus in blood *V* at constant rate *β* and their death rate *d*. The infected cells, *I*, increase with the new infections at rate *βV*(*t*)*T*(*t*) and die at constant rate *δ*. The virus *V* is produced at rate *p* by each infected cell and is cleared at constant rate *c*. The *ε* term denotes the effectiveness of the anti-viral treatment that decreases viral production from *p* to (1 − *ε*)*p*. The previously established ensemble of ODEs for this model is:

(1a) (1b) (1c) $$\left\{ \begin{array}{l} \frac{dT(t)}{dt}=s-\beta V(t)T(t)-dT(t)\\ \frac{dI(t)}{dt}=\beta V(t)T(t)-\delta I(t)\\ \frac{dV(t)}{dt}=(1-\varepsilon )pI(t)-cV(t). \end{array}\right.$$

From the mathematical perspective, the model is simple and can be solved analytically by appropriate assumptions.

### 2.2. The HCV Age-Based Multiscale Model

**FIGURE 1:**
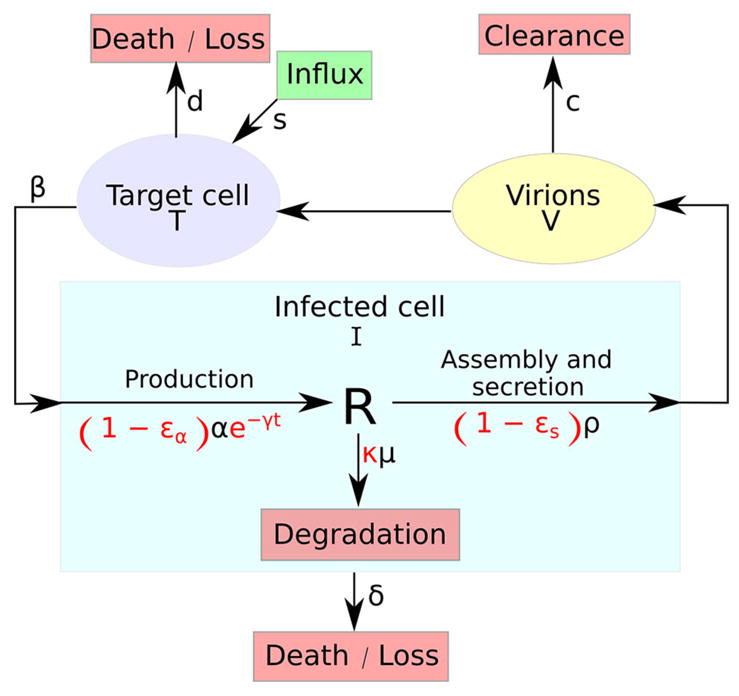
A systematic overview of the multiscale model. The multiscale model accounts for the intracellular HCV RNA (vRNA) replication, *R*, i.e., synthesis, degradation and assembly/secretion with rate parameters *α*, *μ*, and *ρ*, respectively. Treatment (parameters in red) may block vRNA synthesis with effectiveness *ε_α_*, with an additional time-dependent drug-mediated decrease *e*^−^*^γt^* and/or virion assembly/secretion with effectiveness *ε_s_* and/or enhance the degradation rate of vRNA by a factor *κ. T* and *I* represent target and infected cells, respectively, and *V* represents extracellular virus. Target cells are created and die with constant rates *s* and *d* (shown as *D* in the GUI), respectively, and can be infected by virus, *V*, with rate constant *β*. Infected cells, *I*, are lost with rate constant *δ* and virus, *V*, is cleared from blood with rate constant *c*.

The multiscale model of HCV dynamics has been formulated in recent years [25, 41, 43] in order to study HCV dynamics in patients and decide among various treatment options. Figure 1 depicts a systematic overview of the model, which is described by the following partial differential equations (PDEs):

(2a) (2b) (2c) (2d) $$\left\{ \begin{array}{l} \frac{dT(t)}{dt}=s-\beta V(t)T(t)-dT(t)\\ \frac{\partial I(a,t)}{\partial t}+\frac{\partial I(a,t)}{\partial a}=-\delta (a)I(a,t)\\ \frac{dV(t)}{dt}=(1-\varepsilon_{s})\int_{0}^{\infty }\rho (a)R(a,t)I(a,t)\;da-cV(t)\\ \frac{\partial R(a,t)}{\partial t}+\frac{\partial R(a,t)}{\partial a}=(1-\varepsilon_{\alpha })\alpha (a)-\left[\left(1-\varepsilon_{s})\rho (a)+\kappa \mu (a\right)\right]\;R(a,t), \end{array}\right.$$

subject to the boundary conditions *I*(0, *t*) = *βV*(*t*)*T*(*t*), *I*(*a*, 0) = *Ī*(*a*), *R*(0, *t*) = 1, and *R*(*a*, 0) = *R̄* (*a*).

The variable *I*(*a*, *t*) for infected cells, that existed in the standard model as simply *I*(*t*), and the newly introduced variable *R*(*a*, *t*) for intracellular viral RNA (vRNA) both depend on the age of infection *a* and the time duration from therapy initiation *t*. Hence, *a* and *t* are two different times, with the use of partial derivatives in Equations (2b) and (2d). Model parameters of *T*, Equation (2a), and *I*, Equation (2b), are similar to the standard model. The quantity of vRNA *R*, in Equation (2d), depends on its synthesis *α* and its degradation *μ* and secretion from the cell as virus particles *ρ*. The quantity of extracellular virus *V* shown in Equation (2c) depends on the number of assembled and released virions and their clearance rate *c*. A schematic description of the multiscale model is shown in Figure 1.

An important consideration in this model is that the treatment starts after the infection has reached its steady state. The steady states of the different functions are *R̄* (*a*, *t*), *Ī* (*a*, *t*), *V̄* and *T̄*. Given *N*, the total number of virions produced by a cell in its life-span, it was shown in Rong et al. [41] that those values are:

(3) $$N=\frac{\rho (\alpha +\delta )}{\delta (\rho +\mu +\delta )}\;\bar{R}(a,t)=\frac{\alpha }{\rho +\mu }+(1-\frac{\alpha }{\rho +\mu })e^{-(\rho +\mu )a}\;\bar{I}(a,t)=\beta \bar{V}\bar{T}e^{-\delta a}\;\bar{T}=c/(\beta N)\;\bar{V}=(\beta Ns-dc)/(\beta c).$$

Unlike the standard model, three different antiviral effects of therapy can be simulated in the multiscale model. The decrease in viral RNA synthesis is represented by *ε_α_*, the reduction in secretion by *ε_s_* and the increase in viral degradation by *κ* ≥ 1.

Through the method of characteristics, as was derived in Rong et al. [41], an analytical solution was found for the variable *R*(*a*, *t*). The same method was applied to derive a solution for *I*(*a*, *t*). The ensemble of Equations (2) represents the full model. The analytical solutions for *R*(*a*, *t*) and *I*(*a*, *t*) were described as follows:

(4) $$R(a,t)=\left\{ \begin{array}{ll} \frac{\alpha }{\rho +\mu }+\left(1-\frac{\alpha }{\rho +\mu }\right)\;e^{-[\rho +\mu ]a} & a<t\\ \frac{\alpha }{\left(\rho +\mu \right)}+\left(\bar{R}(a-t)-\frac{\alpha }{\left(\rho +\mu \right)}\right)\;e^{-(\rho +\mu )t} & a\geq t \end{array}\right.$$

(5) $$I(a,t)=\left\{ \begin{array}{ll} \beta V(t-a)T(t-a)e^{-\delta a} & a<t\\ \bar{I}(a-t)e^{-\delta t} & a\geq t \end{array}\right.$$

From the system of Equations (2) it can be noticed that computing *V*(*t*) necessitates an integral. If *a* < *t*, in other words the cell age is younger than the time of treatment, i.e., infection occurs after initiation of treatment, the term *I*(*a*, *t*) of the integral in Equation (2) depends itself on *V*(*t*) and *T*(*t*) by consideration of Equation (5). As was shown in Guedj et al., Rong et al., Rong and Perelson [25, 41, 43], this makes the analytical solution for *V*(*t*) approximative.

## 3. MATERIALS AND METHODS

The mathematical difficulties in deriving the long-term approximation, itself imprecise, hinders the generalization to more complex models. Numerical solutions are time consuming unless an efficient method with an adaptive stepsize is implemented, herein the Rosenbrock method [47]. We present how it derives from the Runge-Kutta family methods and its implementation for solving the model shown in Equation 2.

Runge-Kutta methods rely on computing the weighted average of a small increment from the starting position [48]. Those weights, *b_i_*, are predetermined. An advantage of these methods is that because of the use of Taylor series there exist a different set of weights $b_{i}^{*}$, also known, that allow to compute directly the approximation error. The number of terms needed to compute the next value is called the order *S*.

### 3.1. A General Introduction to the Rosenbrock Method

In this section we wish to approximate the function *y*(*t*) for solving the differential equation $\frac{dy}{dt}=f(t,y)$, where *f* (*t*, *y*) is known. Finite-difference methods of higher order are used. Given a known value of *y* at time step *n*, *y_n_*, compute the value at the next time step *y_n_*_+1_ by means of *f* (*t_n_*, *y_n_*).

#### 3.1.1. Explicit Runge-Kutta

The explicit Runge-Kutta family is a generalization of the Euler method to higher order:

(6) $$y_{n+1}=y_{n}+h\sum_{i=1}^{S}k_{i}b_{i}$$

(7) $$t_{n+1}=t_{n}+h,$$

where

(8) $$k_{i}=f(t_{n}+c_{i}h,\;y_{n}+h\sum_{j=1}^{i-1}a_{ij}k_{j})$$

and *a_ij_*, *b_i_*, *c_i_* are pre-determined constants. They are usually displayed in a Butcher tableau as in Table 1. It is important to note the limits of the summation in Equation (8), from 1 to *i* − 1. In this manner, it is straight forward to compute *k_i_* at every step. A drawback of this method is the lack of stability for stiff problems [49]. The implicit Runge-Kutta methods are offering a solution to the instability problem.

#### 3.1.2. Implicit Runge-Kutta

The implicit Runge-Kutta methods are themselves a more stable version of the explicit Runge-Kutta family [48], which is recommended to be used in stiff problems. The equations extend to:

(9) $$y_{n+1}=y_{n}+h\sum_{i=1}^{S}k_{i}b_{i}$$

(10) $$t_{n+1}=t_{n}+h$$

(11) $$k_{i}=f(t_{n}+c_{i}h,\;y_{n}+h\sum_{j=1}^{S}a_{ij}k_{j})$$

and as before, *a_ij_*, *b_i_*, *c_i_* are pre-determined constants. The main difference lies in Equation (11) where the summation is taken from 1 to *S*. The difference becomes obvious when looking at the updated Butcher tableau (Table 2). With the table now full, at each iteration a system of *S* equations with *S* unknowns needs to be solved.

#### 3.1.3. The Rosenbrock Method

As shown in Rosenbrock [47] a special case of the implicit Runge-Kutta methods is when all of the values in the Butcher tableau in the upper triangular matrix above the main diagonal are null and all the diagonal elements equal to a single value *gamma* (i.e., ∀*i a_ii_* = *γ*, Table 3). The value of *k_i_* shown in Equation (11) can now be simplified as follows:

(12) $$k_{i}=f(t_{n}+c_{i}h,\;y_{n}+h\sum_{j=1}^{i}a_{ij}k_{j}).$$

Additional substitutions [50, 51] allow to rewrite the problem as follows:

(13) $$y_{n+1}=y_{n}+h\sum_{i=1}^{S}g_{i}b_{i}$$

(14) $$(I/h\gamma -J_{n})g_{i}=f\;\left(y_{n}+\sum_{j=1}^{i-1}\alpha_{ij}g_{j}\right)+\frac{1}{h}\sum_{j=1}^{i-1}\gamma_{ij}g_{j},$$

where *J_n_* is the Jacobian of *f* at step *n*. To evaluate the value *y_n_*_+1_ we now need to compute the values of the *g_i_*’s. One can notice how the left term of the equation contains a matrix with the term *J_n_*, which is the same for all *g_i_*’s. This is one of the key strategies of this method since to solve for *g_i_* there is only one matrix to invert per time step. It will be taken advantage of by performing an LU decomposition, as shown in the continuation.

Additionally the error *e_n_*_+1_ can be computed as $e_{n+1}:=\sum_{i=1}^{S}g_{i}b_{i}^{*}$ where the $b_{i}^{*}$’s are constants [50]. Given a threshold on the error, the strategy to decide if the step size must be increased or decreased consists in computing the value Δ*_n_*_+1_ :=*e_n_*_+1_/(TOL×(*y_n_* + *f* (*t_n_*, *y_n_*) + 10^−30^)). If Δ*_n_*_+1_ is smaller than 1 then the step size can be increased, else it must be decreased [52]. A good strategy in the latter case is to recompute the *n* + 1 value but with a smaller step size.

### 3.2. The Multiscale Model Solution

Since serum HCV RNA is stable in chronic HCV subjects, we assume that the model is at steady state before the start of treatment, as shown in Equation (3) and discussed in Rong et al. [41]. While individuals with recent HCV infection might not be at steady state at initiation of DAA therapy [3], it may be feasible to assume a quasi-steady state with lower pre-treatment HCV RNA levels, as observed in some subjects, which could be adjusted by changing model parameters.

We implemented the multiscale model with the Rosenbrock method of order *S* = 4. We define:

(15) $$f(t_{n},y_{n}):=\left(\begin{array}{l} \frac{dT}{dt}(t_{n},y_{n})\\ \frac{dV}{dt}(t_{n},y_{n}) \end{array}\right)=\left(\begin{array}{c} s-dT_{n}-\beta V_{n}T_{n}\\ (1-\varepsilon_{s})\int_{0}^{\infty }\rho (a)R(a,t)I(a,t)\;da-cV_{n} \end{array}\right),$$

where $y_{n}=\left(\begin{array}{c} T_{n}\\ V_{n} \end{array}\right)$ and

(16) $$J_{n}:=\left(\begin{array}{cc} \frac{\partial \frac{dT}{dt}}{\partial T} & \frac{\partial \frac{dT}{dt}}{\partial V}\\ \frac{\partial \frac{dV}{dt}}{\partial T} & \frac{\partial \frac{dV}{dt}}{\partial V} \end{array}\right)\;=\left(\begin{array}{cc} -d-\beta V_{n} & -\beta T_{n}\\ 0 & -c \end{array}\right).$$

We note that the term $(1-\varepsilon_{s})\int_{0}^{\infty }\rho (a)R(a,t)I(a,t)\;da$ is constant at each iteration and is computed between each one once. We can now explicitly write the values of the constants and describe the whole scheme. The Equations (13) and (14) become:

(17) $$(2\frac{I}{h}-\left(\begin{array}{cc} -d-\beta V_{n} & -\beta T_{n}\\ 0 & -c \end{array}\right))\;g_{1}=f\;\left(t,y_{n}\right)$$

(18) $$(2\frac{I}{h}-\left(\begin{array}{cc} -d-\beta V_{n} & -\beta T_{n}\\ 0 & -c \end{array}\right))\;g_{2}=f\;\left(t,y_{n}+2g_{1}\right)+\frac{1}{h}\;\left(-8g_{1}\right)$$

(19) $$(2\frac{I}{h}-\left(\begin{array}{cc} -d-\beta V_{n} & -\beta T_{n}\\ 0 & -c \end{array}\right))\;g_{3}=f\;\left(t,y_{n}+\frac{48}{25}g_{1}+\frac{6}{25}g_{2}\right)+\frac{1}{h}\;\left(\frac{372}{25}g_{1}+\frac{12}{5}g_{2}\right)$$

(20) $$(2\frac{I}{h}-\left(\begin{array}{cc} -d-\beta V_{n} & -\beta T_{n}\\ 0 & -c \end{array}\right))\;g_{4}=f\;\left(t,y_{n}+\frac{48}{25}g_{1}+\frac{6}{25}g_{2}\right)+\frac{1}{h}\;\left(\frac{-112}{125}g_{1}+\frac{-54}{125}g_{2}+\frac{-2}{5}g_{3}\right)$$

(21) $$y_{n+1}=y_{n}+\frac{19}{9}g_{1}+\frac{1}{2}g_{2}+\frac{25}{108}g_{3}+\frac{125}{108}g_{4}$$

(22) $$e_{n+1}=\frac{17}{54}g_{1}+\frac{7}{36}g_{2}+0g_{3}+\frac{125}{108}g_{4}$$

Note that the computations of *g*_3_ in Equation (19) and *g*_4_ in Equation (20) use the same value of *f*, which implies that it needs to be computed only once. The error term also disregards the value of *g*_3_.

The scheme for one iteration is shown in Algorithm 1. An important observation is how the error term *e* is computed. It derives only from the error induced by the Rosenbrock iteration, not the computation of the integral term $(1-\varepsilon_{s})\int_{0}^{\infty }\rho (a)R(a,t)I(a,t)\;da$.

The system $(I/h\gamma -J_{n})g_{i}=f\;\left(y_{n}+\sum_{j=1}^{i-1}\alpha_{ij}g_{j}\right)+\frac{1}{h}\sum_{j=1}^{i-1}\gamma_{ij}g_{j}$ needs to be solved for four different values of *i*. Since the left hand matrix is constant, it is decomposed into its LU decomposition once. The Jama package [53] is used to perform the LU decomposition for solving the system in a highly efficient manner.

## 4. RESULTS AND DISCUSSION

A preliminary version of the Rosenbrock method was first implemented in Python3 using the SciPy library, freely available at http://www.cs.bgu.ac.il/~dbarash/HCVnumerics, and then converted to Java for the purpose of developing a user-friendly simulator with a graphical user interface (GUI) that is freely available at http://www.cs.bgu.ac.il/~dbarash/HCVsimulator. Aside of the Rosenbrock method, the default implementation in SciPy of an ordinary differential solver leverages ODEPACK [54] and is referred to as the canned solver *Default*. For this entire section, unless stated otherwise, we used the parameters from Rong et al. [41] and shown in Table 4. The parameters that are changed through the results are the number of days, the size *h* of the steps for the ODEs, and the size *h_a_* of the steps for computing the integral. In the case of the Rosenbrock method that utilizes an adaptive stepsize, we bound the stepsize by *h* as minimum and *h_a_* as maximum.

### 4.1. Short-Term Approximation Holds for Half a Day

**FIGURE 2:**
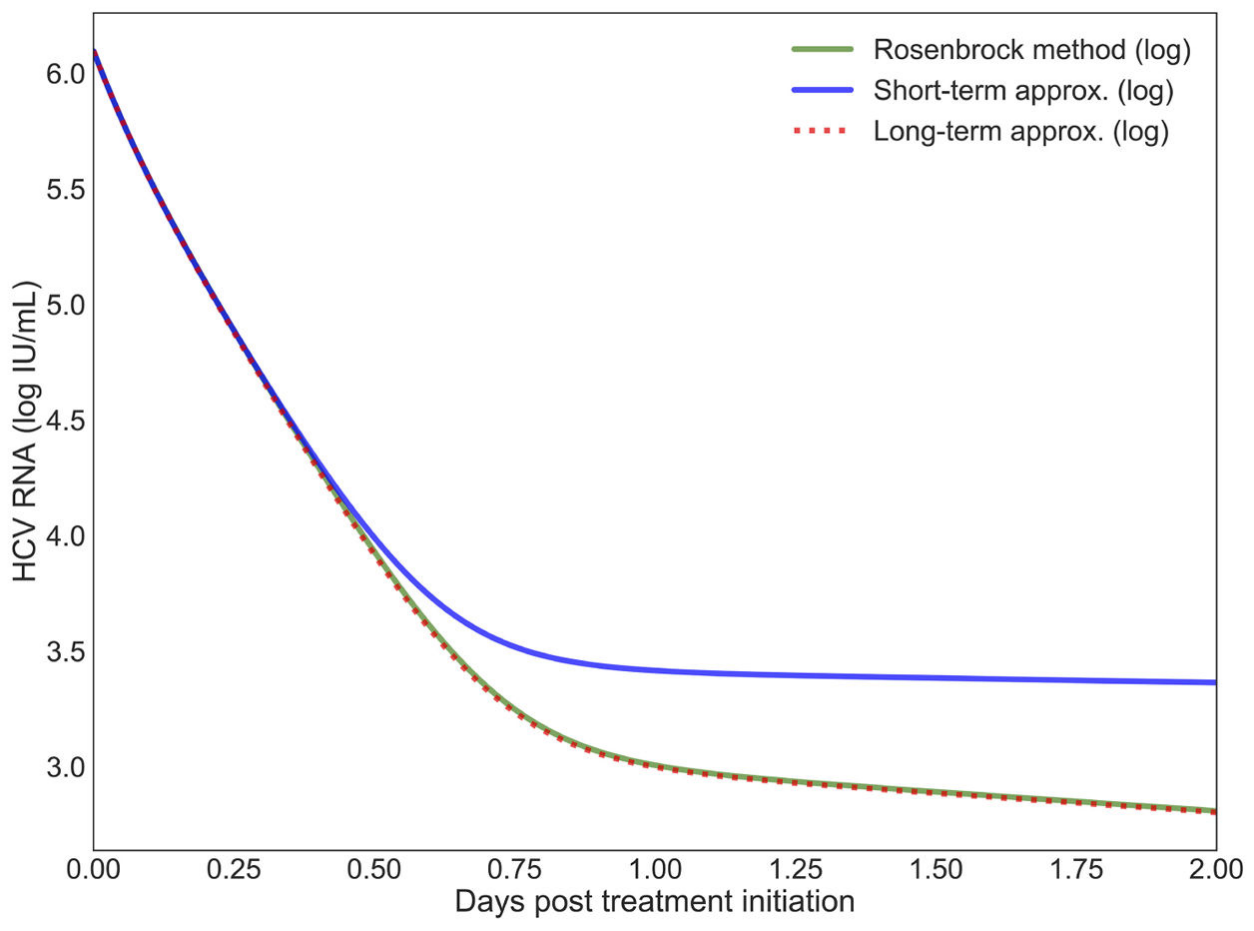
The log values of the short-term and long-term approximations developed in Rong et al. [41] are shown compared to the PDE solution using the Rosenbrock method. Model parameters are described in Table 4 with *h* = 0.001, *h_a_* = 0.01.

The short-term approximation [41] is shown in Figure 2 in blue. The results are shown for only 2 days since the behavior is smooth and conserved on longer time scales in agreement with previous results (Figure 2A in [41]). It is clear that after 12 h the value converges far from the PDE solution. This is expected since the effect of the treatment on the infection rate is not taken into account. In practice, most simulations are valuable for more than half a day and are focused on the long-term approximation.

### 4.2. Long-Term Approximation Underestimates PDE

**FIGURE 3:**
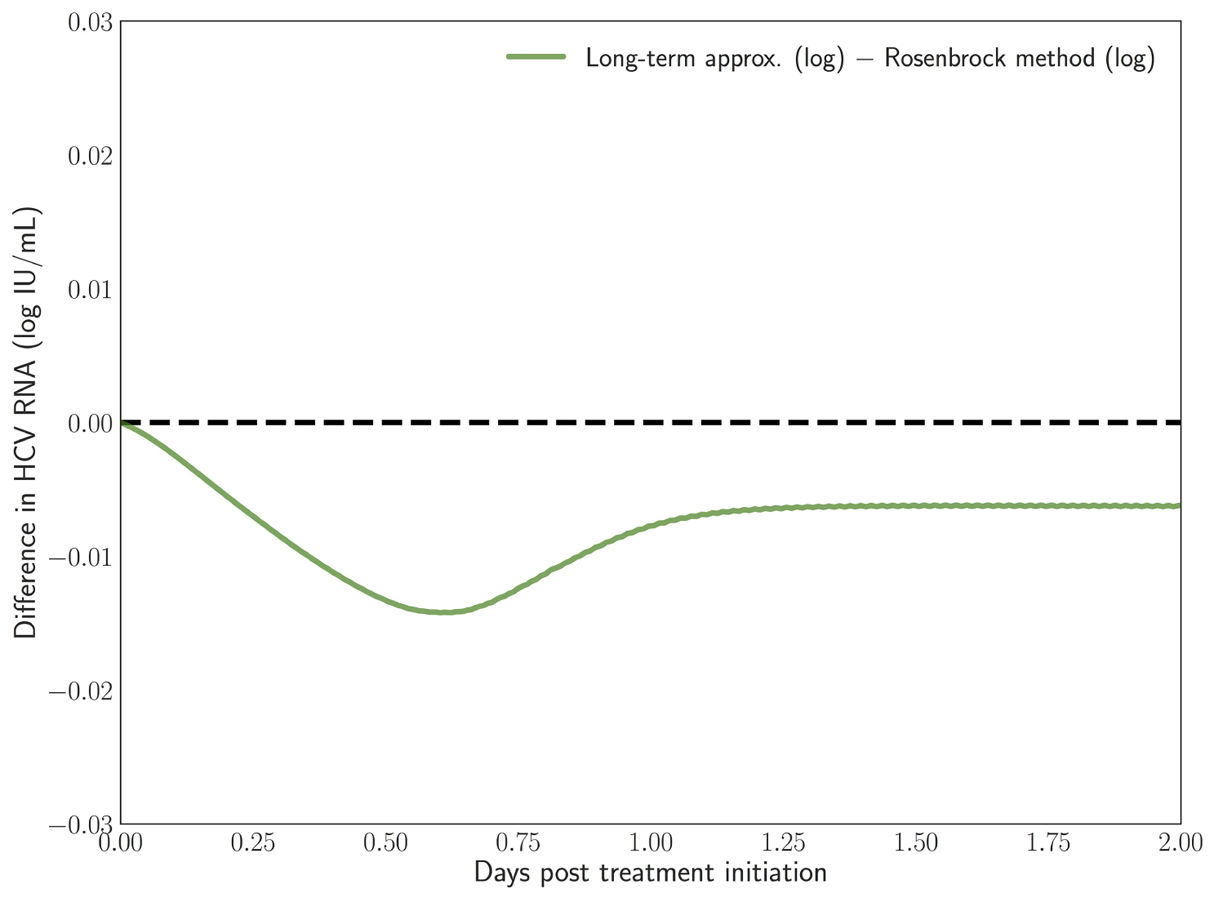
The difference between the long-term approximation, developed in Rong et al. [41], and the PDE solution using the Rosenbrock method described herein is computed. Model parameters are as in Figure 2. The method parameters are *h* = 0.001, *h_a_* = 0.01.

In Rong and Perelson [43] it is hypothesized that the long-term approximation is an **underestimate** of the PDE model solution since some infection events are being ignored. However, with realistic parameters characteristic of potent therapy, the difference between them is very small. Figure 3 depicts the result of our scheme, measuring the difference between the long-term approximation and the solution of the multiscale PDE model (Figure 2B in [41]). We show that the long-term approximation is converging and we are indeed obtaining that the long-term approximation is an underestimate of the PDE model solution (as the difference between the long term approximation and the solution of the PDE model is negative). The difference of less than 0.01 that we are obtaining is very small compared to the y-axis units shown in Figure 1 of Rong and Perelson [43]. Therefore, it is possible to view in Figure 3 this small effect that is an improvement over what was inferred in Rong and Perelson [43] regarding the long-term approximation. This finding is reiterated here for completeness and is referred to below in the sub-section about the Rosenbrock method.

### 4.3. The Model Equations Are Stiff

An important distinction of the age-based PDE HCV model equations is their stiffness, or how they can be numerically unstable even with a small stepsize. We have verified that with small time-steps the results are similar for all methods. But as we increase the size of the steps, the instability of the equations can be observed over 20 days. All explicit methods such as the standard version of Runge-Kutta quickly diverge from the solution with small time-steps. On the other hand an implicit Runge-Kutta method tends to the correct solution and the Rosenbrock method does so efficiently as reported below.

### 4.4. The Rosenbrock Method Is Efficient and Stable

As previously mentioned, the integral term $(1-\varepsilon_{s})\int_{0}^{\infty }\rho (a)R(a,t)I(a,t)\;da$ impedes the use of adaptive stepsize when a canned solver is used. Such methods are essential to allow fast computations of complex models like ours. We present in Table 5 the number of iterations computed using the Rosenbrock method, shown in the previous section, in comparison with the default canned method for three different time step values of *h* and *h_a_*. With the smallest time step, the Rosenbrock method is more than five times as efficient as the default canned method, giving results of similar accuracy. This is crucial since the methods with fixed time step can take up to tens of seconds per day of simulation with *h* = 0.001 and *h_a_* = 0.01. For most research needs, simulations are expected to be performed numerous times for various instances and parameter values. Under those conditions a five-fold increase in speed, through the decrease in the number of iterations, provides a much needed advantage when testing large number of parameters over long time periods. Interestingly with the highest value of *h*, the Rosenbrock method takes additional iterations. This is due to the greediness of the time step adjustment, which when increased too quickly and thereby inducing a large error backtracks and starts again with a smaller value.

Simpler methods than Rosenbrock exist with adaptive stepsize, such as Dormand–Prince [48], Fehlberg (RKF) [55] and Cash-Karp (RKCK) [56]. These are explicit methods that can be easily implemented but are vulnerable to the stiffness of the equations, while Rosenbrock is an implicit method that is indeed found to be more stable. In the following comparisons we demonstrate that the Rosenbrock method is more efficient than the Runge-Kutta 4th order method and at the same time it is more stable than both the Runge-Kutta 4th order and the Dormand-Prince methods.

First, a comparison of the computing time between the Rosenbrock method and the other methods mentioned above is reported in Table 6 with starting time step values of *h* = 0.001 and *h_a_* = 0.01 for 14 days of simulation. Each run with a certain method was repeated 10 times, after which the mean and standard deviation was taken. The Rosenbrock method is over 8 times faster than the standard Runge-Kutta 4th order method, which is a non-adaptive time step method. While Dormand–Prince is an adaptive time step method that is even faster than Rosenbrock, as will be shown below, it is an explicit method that does not always converge to the correct solution. The fastest time of execution for the Dormand-Prince method is not surprising since an explicit method is easier to evaluate computationally than an implicit method, in particular because there is no need for matrix inversions.

**FIGURE 4:**
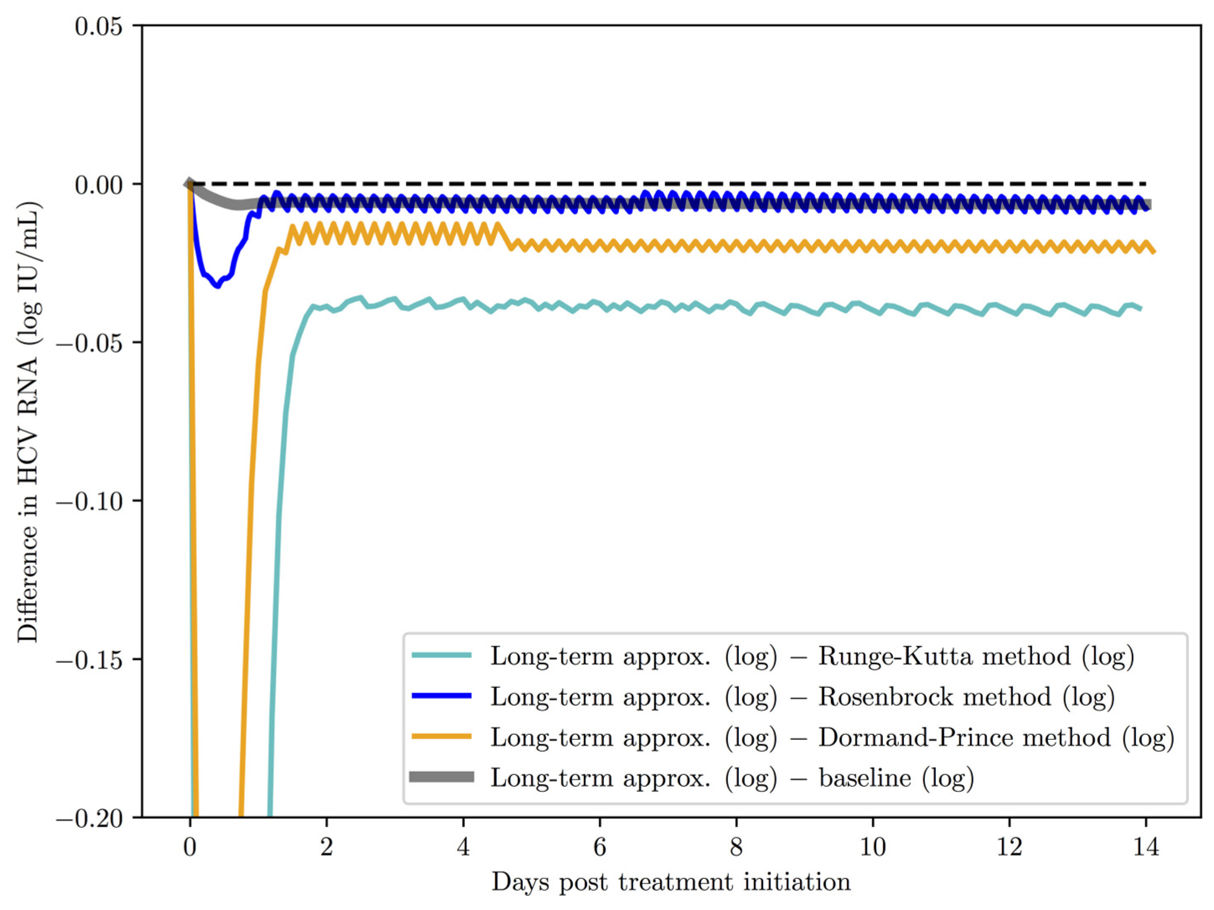
A comparison of the Rosenbrock method with other methods over 14 days of simulation with starting time steps of *h* = 0.1 and *h_a_* = 0.1. The baseline (the default implementation in the SciPy library of an ODE solver) was computed with time steps of *h* = 0.001 and *h_a_* = 0.01 since with these time step values and smaller, as expected, all methods converged to the solution of the baseline.

Second, a comparison of the methods behavior over 14 days of simulation is presented in Figure 4. All the methods were started with time steps of *h* = 0.1 and *h_a_* = 0.1. The baseline (the default implementation in the SciPy library of an ODE solver) was computed for *h* = 0.001 and *h_a_* = 0.01 since those time step values are small enough to ensure that all methods converge to the same solution. The advantage of the Rosenbrock method in terms of stability and convergence to the correct solution is clearly noticed.

Finally, we also report in Table 7 the sensitivity of the Rosenbrock method. We compute the changed value of the result after 2 days of simulation given a variation of ±10% on 10 parameters. We notice that most of the perturbations are symmetrical. We omit *ε_α_* and *ε_s_* since they effectively determine the fraction of *α* and *ρ* used in the model. The results (Table 7) indicate that the Rosenbrock method is sufficiently robust for our needs.

### 4.5. The Multiscale Model Simulator

**FIGURE 5:**
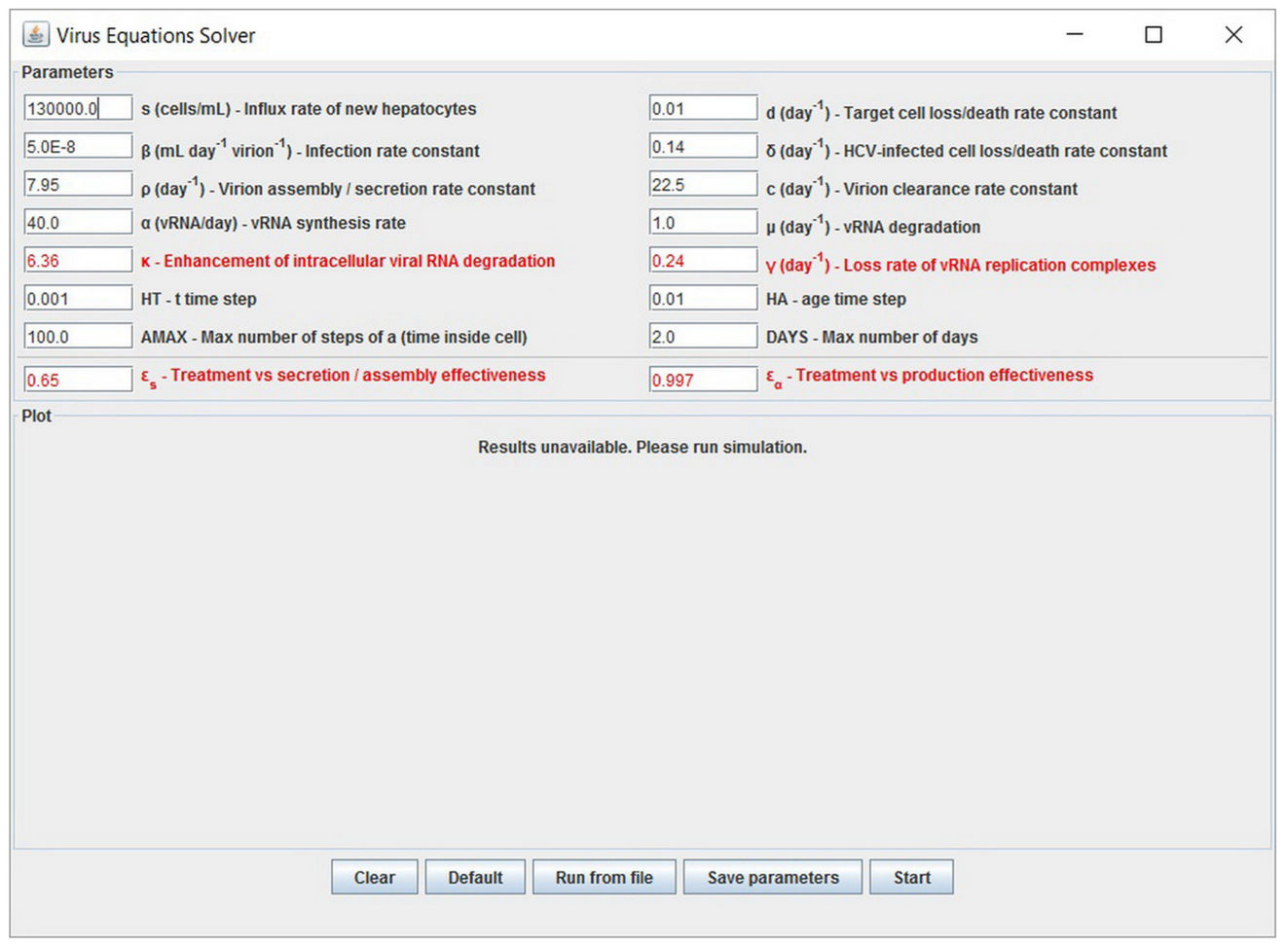
Graphical user interface (GUI). The displayed values are the default options and can be modified. At the bottom are the options to add experimental data and parameters values from a file. Note that setting the four parameters *ε_α_*, *ε_s_*, *κ* and *γ* (red fonts) to 0 will keep the system in the pre-treatment steady state. An option to export the parameters is also available.

**FIGURE 6:**
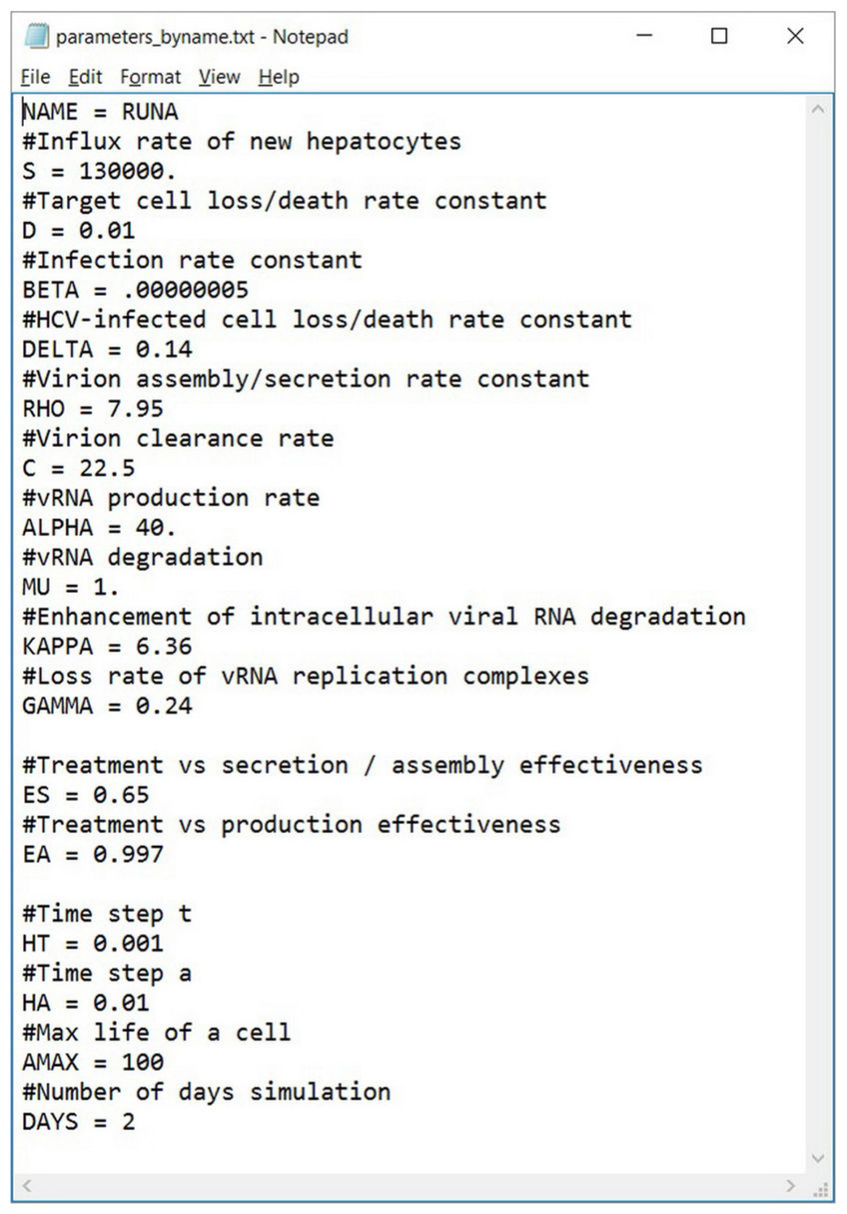
An example of a parameters file that can be automatically uploaded. Lines starting with a # are comments (ignored). This file can also be exported from our software, allowing users to share them easily.

**FIGURE 7:**
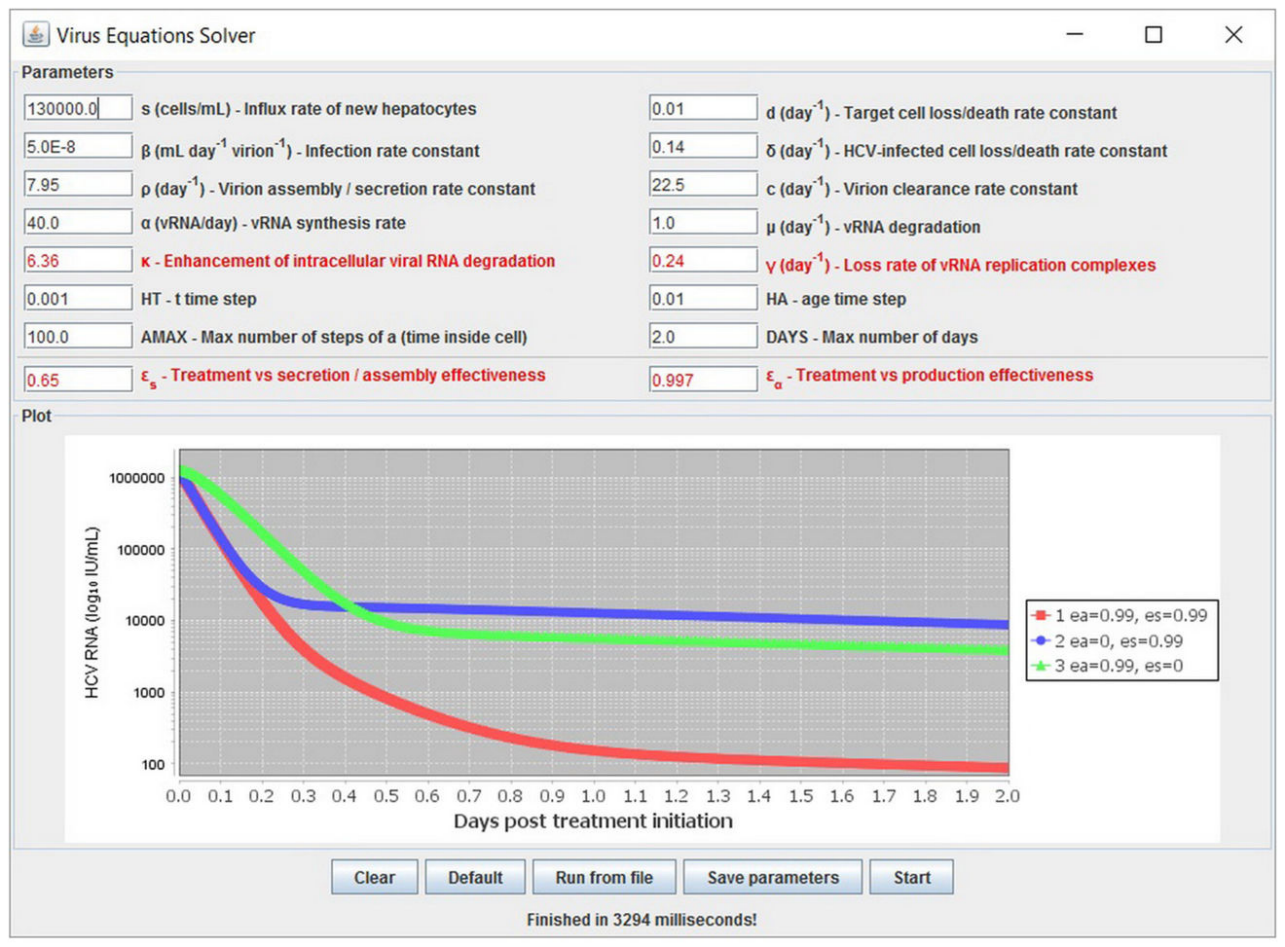
Several simulations can be displayed simultaneously. Three treatment scenarios are plotted with similar results as previously shown in Rong et al. [41].

**FIGURE 8:**
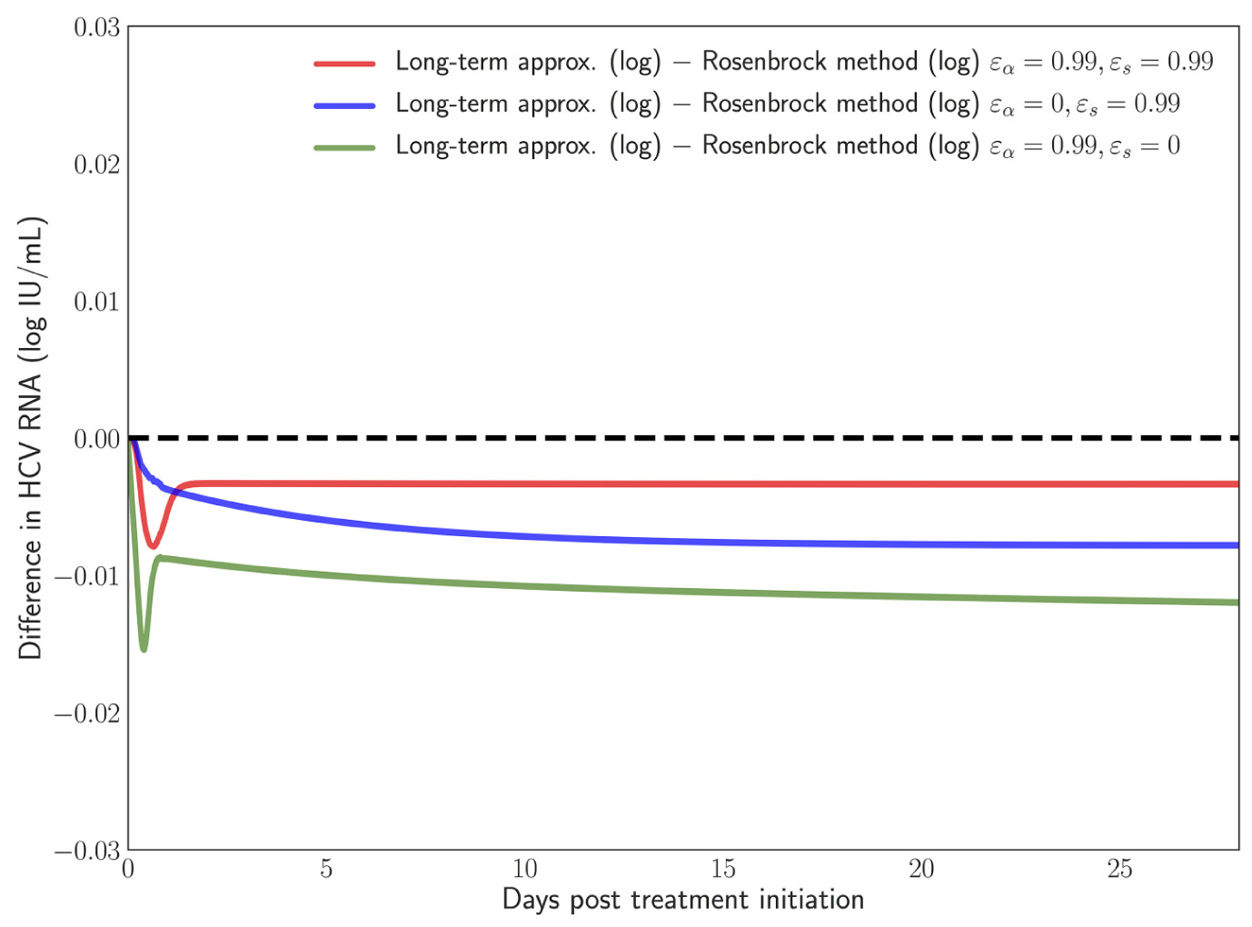
Difference in the log values between the long-term approximation [41] and the Rosenbrock method when choosing *ε_α_* and *ε_s_* from (0, 0.99). One can notice how even at the longer treatment duration the difference remains minimal and mostly constant.

We have developed a user-friendly simulator with a GUI for the multiscale model (Figure 5) that is freely available at http://www.cs.bgu.ac.il/~dbarash/HCVsimulator. The parameters file input is shown in Figure 6. For illustration, two example simulations are provided, one over 2 days (Figure 7) and one over 28 days (Figure 8). In Figure 7 we show how different parameter ensembles can be displayed simultaneously. In that case we chose *ε_α_* and *ε_s_* to be 0 or 0.99, resulting in three curves. We can observe that increasing the *ε_s_* parameter, which decreases the assembly/secretion of intracellular virions, has a strong short term effect that tempers quickly. In contrast the reduction in the synthesis of vRNA, modeled by an increase of the parameter *ε_α_*, shows a slower but more efficient effect that exhibits stronger results after half a day. Combining both factors shows a multiphasic viral decline which was observed under treatment with HCV NS5A inhibitors [25]. In Figure 8 we present the difference between the long-term approximation and the Rosenbrock method for those three sets of parameters over longer (than 2 days) treatment durations (e.g., 28 days). Although the highest error is present when *ε_s_* = 0, the error remains below a tenth of the differences of the log values. Thus, in all cases the trends from the first 2 days continue in the next 4 weeks and the error grows minimally.

## 5. SUMMARY AND CONCLUSIONS

Modeling intracellular viral RNA dynamics within infected cells is becoming an improtant mean for considering various curative treatment options. A viral dynamic model that considers intracellular viral RNA replication, namely an age-structured PDE multiscale model, has been recently put forth to study viral hepatitis dynamics during antiviral therapy [25, 41, 43]. This type of model is more complicated to solve than previous ODEs models. The seminal works that introduce the age-based multiscale model predominantly use analytical approximations, with some numerical solutions that are either based on simple first-order methods or canned solvers [25, 41, 43]. Neither of these numerical solutions are satisfactory for realistic simulations of several days of infection and therapy in terms of accuracy, efficiency and stability.

We first showed that the long-term approximation is an underestimate of the PDE model solution, which was anticipated in Rong and Perelson [43] because some infection events are being ignored in this analytical approximation. We then observed that the governing differential equations are stiff and therefore advanced numerical methods are needed. Methods with fixed stepsize or alternatively, the use of canned routines, do not offer a comprehensive solution to the model and are limited in scope. For example, in the multiscale model, there is an integral term that needs to be computed at each time step depending on previous iterations and this is inadequately done by canned solvers. Methods with adaptive stepsize offer a considerable improvement for realistic simulations. Having previously investigated these methods, we found the Rosebrock method to be the most accurate and efficient among adaptive stepsize methods for this model. We provide a simulator based on the Rosenbrock method with a user-friendly GUI that is freely available at http://www.cs.bgu.ac.il/~dbarash/HCVsimulator.

Future work from the numerical standpoint could potentially include a more comprehensive treatment of the associated integral. In particular the adaptive stepsize techniques rely on the evaluation of the error produced at each evaluation of the ODE equations. Therefore, the error produced by the integral itself is never taken into account. Including that term would allow to relax the restrictions on the stepsize increase and potentially decrease further, beyond what is observed in Table 5 as least number of iterations, the number of iterations that are required to achieve a sufficient accuracy.

The Rosenbrock method implementation provided here can be generalized to potentially assist in understating treatment failure due to drug resistance by the expansion of the age-based model to include viral strains [57]. It could also be used to explore age-based models that include further aspects of intracellular HCV life cycle such as translation positive-strand HCV RNA and the synthesis of positive and negative-strand HCV RNA [58]. Finally, since the current age-based HCV multiscale model is a successful milestone but still fails to predict cure in some DAA-treated patients [59–61], further model modification will be needed [38, 39] and future developments would necessitate some updates in the numerical method. While such models become quickly overwhelming to solve analytically, an extension of the presented method is straightforward. The simulator provided here based on the Rosenbrock method can be modified accordingly and be developed further to accommodate the modelers needs.

## Figures and Tables

**TABLE 1:** Butcher tableau for explicit Runge-Kutta, missing values are null as the *c*_1_ row.

$$\begin{array}{lllll} c_{2} & a_{21} & \; & \; & \;\\ \vdots & \vdots & \ddots & \; & \;\\ c_{S} & a_{S1} & \cdots & a_{S,S-1} & \;\\ \; & b_{1} & \; & \cdots & b_{S}\\ \; & b_{1}^{*} & \; & \cdots & b_{S}^{*} \end{array}$$

**TABLE 2:** Butcher tableau for implicit Runge-Kutta.

$$\begin{array}{llll} c_{1} & a_{11} & \cdots & a_{S1}\\ \vdots & \vdots & \ddots & \vdots \\ c_{S} & a_{S1} & \cdots & a_{SS}\\ \; & b_{1} & \cdot & b_{S}\\ \; & b_{1}^{*} & \cdots & b_{S}^{*} \end{array}$$

**TABLE 3:** Butcher tableau for Rosenbrock, missing values are null.

$$\begin{array}{llll} c_{1} & \gamma & \; & \;\\ \vdots & \vdots & \ddots & \;\\ c_{S} & a_{S1} & \cdots & \gamma \\ \; & b_{1} & \cdots & b_{S}\\ \; & b_{1}^{*} & \cdots & b_{S}^{*} \end{array}$$

**TABLE 4:** The parameters of the model used in all simulations, taken from Rong et al. [41].

Parameter	Value	Parameter	Value
*s*	13,0000 cells/mL	*β*	0.00000005 mL day^−1^ virion^−1^
*d*	0.01 day^−1^	*δ*	0.14 day^−1^
*κ*	6.36	*c*	22.5 day^−1^
*α*	40 day^−1^	*ρ*	7.95 day^−1^
*μ*	1 day^−1^	*γ*	0.24 day^−1^
*ε_s_*	0.65	*ε_α_*	0.997

**TABLE 5:** Number of iterations taken by the Rosenbrock method given time step values *h* and *h_a_* compared to the number of iterations taken with the canned method (*Default*).

*h*	*h_a_*	Rosenbrock	*Default*
0.001	0.01	343	2,000
0.01	0.1	112	200
0.1	1	23	20

**TABLE 6:** Efficiency comparison of the Rosenbrock method with other methods.

Method	Average time (sec)	Standard deviation (sec)
Rosenbrock	78.68	1.16
Runge–Kutta 4th order	687.51	17.72
Dormand–Prince	46.51	1.26
Default	688.73	18.67

The computing time in seconds were taken by the various methods for 14 days of simulation, averaged over 10 repetitions per each method. The starting time step values were h = 0.001 and h_a_ = 0.01 and the runs were performed on a standard PC.

**TABLE 7:** Sensitivity of the Rosenbrock method.

Parameter	Decreased	Increased	Parameter	Decreased	Increased
*s*	0.88%	1.12%	*β*	0.98%	1.01%
*d*	1.02%	0.98%	*δ*	1.16%	0.87%
*κ*	1.08%	0.93%	*c*	1.13%	0.89%
*α*	0.88%	1.12%	*ρ*	0.93%	1.07%
*μ*	1.08%	0.93%	*γ*	1.05%	0.96%

Each of the 10 parameters was decreased and increased by 10% of its original value. For each we present the ratio of the result after 2 days over the result with the original value. We omit ε_α_ and ε_s_ since they effectively determine the fraction of α and ρ used in the model.

**Algorithm 1:** General Rosenbrock Scheme. TOL was set to 10^−4^.